# In-depth analysis of the treatment effect and synergistic mechanism of TanReQing injection on clinical multi-drug resistant *Pseudomonas aeruginosa*

**DOI:** 10.1128/spectrum.02726-23

**Published:** 2024-02-28

**Authors:** Dongying Li, Yueyi Li, Jingyi Wang, Weifeng Yang, Kaiyu Cui, Renjing Su, Lu Li, Xing Ren, Xianyu Li, Yi Wang

**Affiliations:** 1 Beijing Key Laboratory of Traditional Chinese Medicine Basic Research on Prevention and Treatment for Major Diseases, Experimental Research Center, China Academy of Chinese Medical Sciences, Beijing, China; Yangzhou University, Yangzhou, China

**Keywords:** *Pseudomonas aeruginosa*, clinical multidrug-resistant, MALDI-TOF MS, overcoming resistance, TanReQing injection

## Abstract

Antibiotic resistance is a recognized and concerning public health issue. Gram-negative bacilli, such as *Pseudomonas aeruginosa* (*P. aeruginosa*), are notorious for their rapid development of drug resistance, leading to treatment failures. TanReQing injection (TRQ) was chosen to explore its pharmacological mechanisms against clinical multidrug-resistant *P. aeruginosa* (MDR-PA), given its antibacterial and anti-inflammatory properties. We revealed the expression of proteins and genes in *P. aeruginosa* after co-culture with TRQ. This study developed an assessment method to evaluate clinical resistance of *P. aeruginosa* using MALDI-TOF MS identification and Biotyper database searching techniques. Additionally, it combined MIC determination to investigate changes in MDR-PA treated by TRQ. TRQ effectively reduced the MICs of ceftazidime and cefoperazone and enhanced the confidence scores of MDR-PA as identified by mass spectrometry. Using this evaluation method, the fingerprints of standard *P. aeruginosa* and MDR-PA were compared, and the characteristic peptide sequence (Seq-PA No. 1) associated with flagellum was found. The phenotypic experiments were conducted to confirm the effect of TRQ on the motility and adhesion of *P. aeruginosa*. A combination of co-immunoprecipitation and proteome analysis was employed, and 16 proteins were significantly differentially expressed and identified as potential candidates for investigating the mechanism of inhibiting resistance in *P. aeruginosa* treated by TRQ. The candidates were verified by quantitative real-time PCR analysis, and TRQ may affect these core proteins (MexA, MexB, OprM, OprF, OTCase, IDH, and ASL) that influence resistance of *P. aeruginosa*. The combination of multiple methods helps elucidate the synergistic mechanism of TRQ in overcoming resistance of *P. aeruginosa*.

**IMPORTANCE:**

*Pseudomonas aeruginosa* is an opportunistic pathogen closely associated with various life-threatening acute and chronic infections. The presence of antimicrobial resistance and multidrug resistance in *P. aeruginosa* infections significantly complicates antibiotic treatment. The expression of β-lactamase, efflux systems such as MexAB-OprM, and outer membrane permeability are considered to have the greatest impact on the sensitivity of *P. aeruginosa*. The study used a method to assess the clinical resistance of *P. aeruginosa* using matrix-assisted laser desorption ionization time of flight mass spectrometry identification and Biotyper database search techniques. TanReQing injection (TRQ) effectively reduced the MICs of ceftazidime and cefoperazone in multidrug-resistant *P. aeruginosa* (MDR-PA) and improved the confidence scores for co-cultured MDR-PA. The study found a characteristic peptide sequence for distinguishing whether *P. aeruginosa* is resistant. Through co-immunoprecipitation and proteome analysis, we explored the mechanism of TRQ overcoming resistance of *P. aeruginosa*.

## INTRODUCTION

*Pseudomonas aeruginosa* is classified as an opportunistic pathogen that can cause a wide range of infections in human. These infections can range from nosocomial infections to those associated with cystic fibrosis, ventilator-associated pneumonia, burns, chronic obstructive pulmonary disease, and even those induced by conditions like COVID-19, among others, all attributed to *P. aeruginosa* in clinical settings (1–3). . The rapid mutation and adaptation of *P. aeruginosa* to develop antibiotic resistance present significant challenges for effective therapy. Patients afflicted with multidrug-resistant (MDR) or extensively drug-resistant strains of *P. aeruginosa* are at higher risk for receiving inadequate initial antimicrobial therapy, leading to further resistance and increased mortality rates (4, 5). Antimicrobial resistance is considered as one of the greatest global health threats of the 21st century. According to epidemiological research, bacterial infections caused by antibiotic resistance claimed the lives of up to 700,000 people annually (6). *P. aeruginosa* is one of the six “ESKAPE” superpathogenic bacteria and has been classified as a “critical” pathogen by the World Health Organization. Innovative and urgent treatments are imperative to confront the challenges posed by this highly resistant bacterium (7).

*P. aeruginosa* exhibits a complex resistance mechanism, including inherent, acquired, and adaptive drug resistance, which allows it to develop resistance to a broad spectrum of antibiotics. Factors such as restricted outer membrane permeability, efflux systems like MexAB-OprM, and antibiotic-inactivating enzymes like β-lactamases, among others, collectively contribute to the intrinsic resistance of *P. aeruginosa* to multiple antibiotics at a high level (8, 9). For these intrinsic resistance mechanisms, the resistance-nodulation-cell division (RND) family efflux pumps (MexAB-OprM, MexCD-OprJ, MexEF-OprN, and MexXY-OprM) play an important role in antibiotic resistance of *P. aeruginosa*, which is one of the MDR efflux pumps. Unlike traditional efflux pumps that usually export a single type of drug, MDR efflux pumps have the capacity to excrete a wide range of drugs (10). The acquired resistance of antibiotics is caused by resistance genes transfer and gene mutations. Additionally, some environmental stimuli and bacterial behaviors (e.g., motility, persister cells, and biofilm formation) can lead to adaptive resistance. These triggers may affect the expression of efflux pumps and AmpC, further contributing to antibiotic resistance (11, 12).

Due to the emergence of MDR strains, conventional antibiotic therapy for *P. aeruginosa* infections has progressively lost its effectiveness. Current treatment strategies for *P. aeruginosa* involve the combined use of different antibiotics and ongoing research into the development of novel antibiotics (3). Furthermore, novel therapeutic approaches such as phage therapy, quorum sensing inhibition, immunotherapy, gene editing therapy, antimicrobial peptides, and vaccine therapies have garnered attention in the field. Nevertheless, it is worth noting that the research and development of new antibiotics and other therapeutic techniques are progressing at a slow and time-consuming pace (13). Therefore, in accordance with the literature, the most cost-effective approach against drug-resistant bacteria is to utilize existing medications to restore the susceptibility of drug-resistant bacteria to antibiotics, coupled with effective management strategies (14). Presently, various combinations of antibiotics and synergists, such as β-lactamase inhibitors, have been reported, showing promising potential for restoring antibiotic susceptibility and reversing resistance in resistant pathogens (15). A strategy combining polymers and antibiotics has been reported to restore the rifampicin resistance phenotype in *Acinetobacter baumannii* (16). The combination strategy has laid the foundation for a novel category of antibiotic adjuvants with the potential to reverse drug resistance.

TanReQing injection (TRQ) is a preparation possessing antibacterial and anti-inflammatory properties and is extensively used in the treatment of respiratory tract infections, the prophase of pneumonia, and other related diseases (17, 18). TRQ has obtained approval from the State Drug Administration of China and is a mixed extract derived from bear gall powder, *Scutellariae radix*, *Forsythiae fructus*, *Lonicerae japonicae flos*, and *Caprae hirci cornu*. The main extracts in TRQ include total cholic acid extract of bear gall powder, total amino acid extract of *Caprae hirci cornu*, baicalin extract of *Scutellariae radix*, water extract of *Lonicerae japonicae flos*, and water extract of *Forsythiae fructus*, with each extract blended in a ratio of 4:2:25:25:50. Several reports have been published on the qualitative and quantitative analyses of the chemical composition of TRQ, detecting 11, 60, and 126 components in TRQ, respectively (19–21). The quantitative determination of baicalin, ursodeoxycholic acid, chenodeoxycholic acid, and total amino acids and the qualitative detection of fingerprints were performed according to the officially used drug standards.

Antibacterial and anti-inflammatory effects of TRQ have been reported in the literature. Furthermore, TRQ has been found to be particularly effective when used in conjunction with antibiotics to combat methicillin-resistant *Staphylococcus aureus* pathogens (22). In addition, TRQ has been demonstrated to influence *Staphylococcus aureus* division, reducing virulence (23), and it significantly reduced the expression of genes regulated by quorum sensing in *P. aeruginosa* (24). *P. aeruginosa* remains a major pathogen causing respiratory tract infections in clinical settings. Several studies indicated that the combination of TRQ with antibiotics had better clinical efficacy in the treatment of pulmonary infections caused by *P. aeruginosa* (25–27). However, the underlying mechanism responsible for this synergy remains unclear.

In clinical studies, the primary methods for identifying bacterial isolates have traditionally relied on phenotypic characteristics, including colony morphology, biochemical reactions, growth patterns in different media, and Gram staining. While these techniques generally provide high accuracy, they can be time consuming and costly (28). Currently, matrix-assisted laser desorption ionization time of flight mass spectrometry (MALDI-TOF MS) is widely used for the identification of various bacteria isolated from clinical samples. This technology enables the rapid identification of bacteria by comparing their fingerprints with those in the reference databases. MALDI-TOF MS has significantly accelerated diagnosis while simultaneously reducing medical expenses. Moreover, several academic groups have proposed innovative solutions to accelerate antimicrobial susceptibility testing (AST) and the detection of bacterial resistance-associated proteins. For instance, they have developed methods for the rapid detection of carbapenemase production using this method (29–32).

To investigate the pharmacological mechanism of TRQ on multidrug-resistant *P. aeruginosa* (MDR-PA), we developed an evaluation method using *P. aeruginosa* as a model microorganism to represent clinically sensitive and resistant strains. This is the first time that MALDI-TOF MS identification has been combined with the minimum inhibitory concentration (MIC) to assess the pharmacological effects of TRQ on MDR-PA. We tried to find the characteristic peptide sequence by comparing the protein fingerprints of standard *P. aeruginosa* and MDR-PA. Additionally, through co-immunoprecipitation (Co-IP) and in-depth proteome analysis of the microorganism (standard *P. aeruginosa* and MDR-PA), we explored the pharmacological mechanism of TRQ. This study reveals the involvement of several key proteins in MDR-PA resistance. After co-cultivation with TRQ, changes in protein expression were observed in MDR-PA, indicating that TRQ has the potential to sensitize MDR-PA.

**Fig 1:**
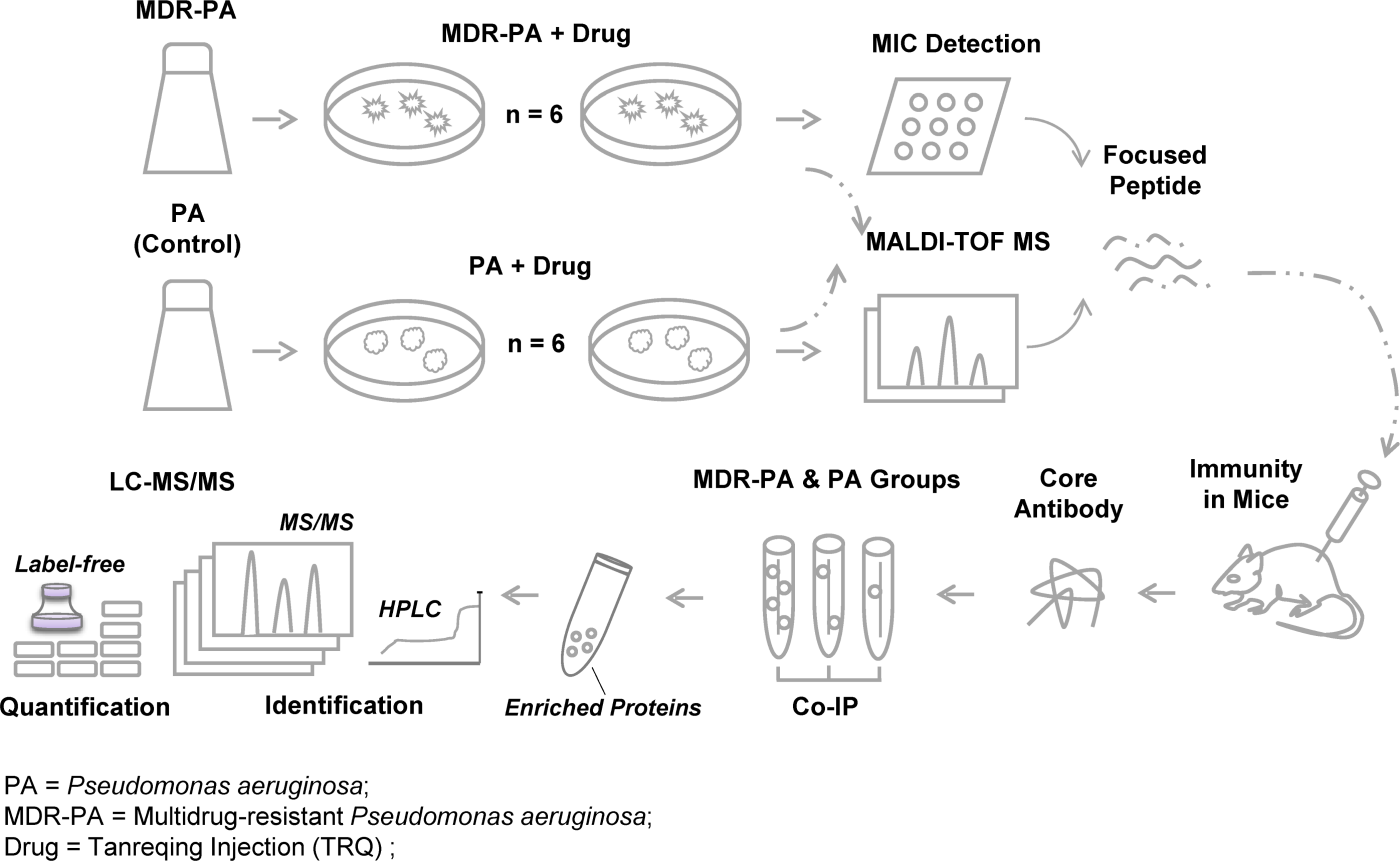
Overview of the experimental workflow. First, the sensitization of TRQ to MDR-PA strains was determined by phenotype experiment. After co-culture of TRQ and MDR-PA/*P. aeruginosa* (PA) for six generations, the MIC values of antibiotics were detected. The confidence scores of co-cultured MDR-PA strains were identified by MALDI-TOF -MS, and the characteristic peptide sequence was found by fingerprint comparison. Second, we obtained antibodies to focused peptides through immunity test of mice and found proteins interacting with immune antibodies by co-immunoprecipitation (Co-IP). Third, proteins for proteome analysis were tryptically digested in solution, desalted using a C18 precolumn, and subjected to LC and Q-Exactive analysis. Then, the peptide mixture was analyzed with online reversed-phase chromatography and mass spectrometry (LC-MS/MS), and a label-free approach was used for the quantitative analysis (proteome analysis). Finally, the gene expression of MDR-PA strain sensitized by TRQ was detected by RT-qPCR and was verified with the change of protein expression.

The overview of the experimental workflow is illustrated in Fig. 1. In brief, standard *P. aeruginosa* and MDR-PA were co-cultured with TRQ for six generations. Changes in MICs of antibiotics were detected, and the confidence scores of co-cultured strains were determined using MALDI-TOF-MS. Characteristic peptide sequence was subsequently identified through mass spectrometry (MS) identification. To focus on the characteristic peptides sequence, antibodies against this peptide were obtained through an immunization test conducted in mice. The mechanism and the core proteins were explored through Co-IP experiments. Proteome analysis was performed by digesting the proteins with trypsin and desalting them with a C18 pre-column. The identification was performed using liquid chromatography-tandem mass spectrometry (LC-MS/MS), and the analysis was conducted using a label-free approach to screen out proteins related to TRQ-sensitized MDR-PA strains. Finally, the gene expression of these relevant proteins was verified through quantitative real-time PCR (RT-qPCR).

## RESULTS

### TRQ decreased the MICs of ceftazidime and cefoperazone against *P. aeruginosa*

**Fig 2:**
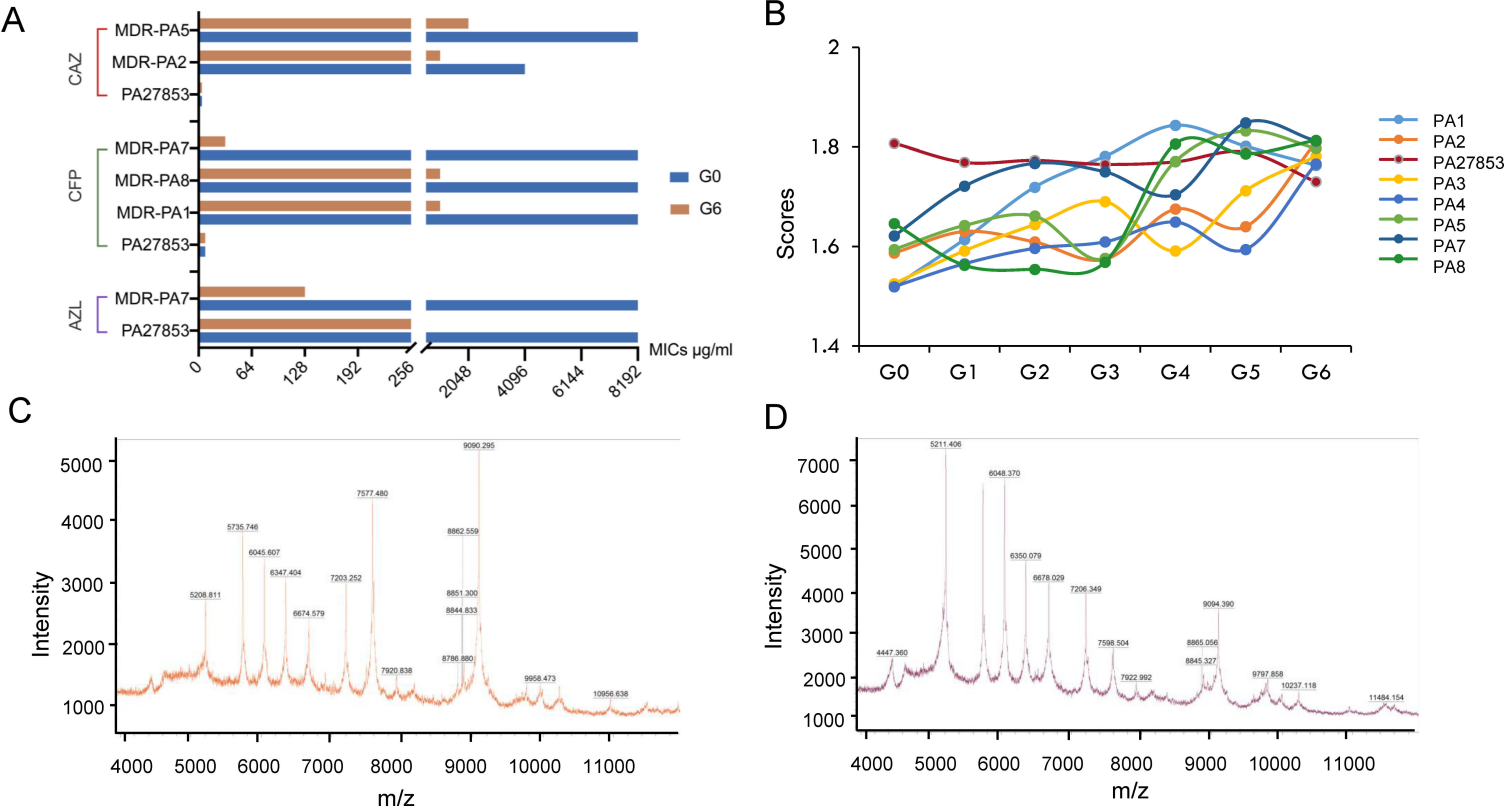
TRQ sensitizes multidrug-resistant *Pseudomonas aeruginosa* (MDR-PA). (**A**) MICs of different antibiotics of G0 and G6 strains after co-culture with TRQ. (**B**) The confidence scores of MDR-PA/*P. aeruginosa* (PA) after co-culture with TRQ by MALDI-TOF-MS. (**C**) The G0 generation of MDR-PA fingerprints by MALDI-TOF-MS. (**D**) The G6 generation of MDR-PA fingerprints by MALDI-TOF-MS.

After co-culturing TRQ with standard *P. aeruginosa* or MDR-PA for six generations, we determined the MICs of different antibiotics. For standard *P. aeruginosa*, the MIC values did not show significant changes. However, for MDR-PA, the MIC of cefoperazone and ceftazidime tended to decrease after co-culture with TRQ (Table S1; Fig. 2A). Before and after co-cultivation with TRQ, the MICs of cefoperazone and ceftazidime against the standard *P. aeruginosa* (PA 27853) strain were consistent at 16 µg/mL and 4 µg/mL, respectively. Notably, the MICs of cefoperazone for various MDR-PA strains decreased significantly: MDR-PA1 from 8,192 μg/mL to 1,024 μg/mL, MDR-PA7 from 8,192 μg/mL to 32 μg/mL, and MDR-PA8 from ≥8,192 μg/mL to 1,024 μg/mL. Similarly, the MICs of ceftazidime against MDR-PA2 decreased from 4,096 μg/mL to 1,024 μg/mL and against MDR-PA5 decreased from 8,192 μg/mL to 2,048 μg/mL. The MIC results suggested that TRQ has an influence on resistance of *P. aeruginosa*.

### TRQ enhanced the confidence scores of MDR-PA

The strains, both co-cultured with and without TRQ, were identified using MALDI-MS and the Biotyper platform. The result showed a gradual increase in the confidence scores for both the original strain and the strains co-cultured with TRQ for six generations (Fig. 2B). The confidence score reflects the match quality of MS peaks between the sample spectrum and the standard fingerprint in the Biotyper database identified by the MALDI-TOF MS. A higher score signifies a better match, and a score within the range of 1.8 to 2.0 represents a perfect match. Notably, the sensitive strain PA27853 exhibited a score within the 1.8–2.0 range, signifying a perfect match. The confidence scores of MDR-PA strains increased from initially below 1.5 to exceed 1.8, progressing after six successive co-cultivation generations. This significant increase in scores and confidence levels suggested that TRQ may sensitize MDR-PA based on the phenotypic results.

Following this, the MALDI fingerprints of different generations of standard *P. aeruginosa* and MDR-PA co-cultured with TRQ from 0 to 6 generation were compared and analyzed using Mascot Engines (Fig. 2C and D). This analysis led to the identification of a significant differential sequence, which was designated as “Seq-PA No. 1” and recognized as a distinctive feature sequence. Meanwhile, it was observed that Seq-PA No. 1 might be associated with the flagella protein of *P. aeruginosa*, which prompted further, in-depth research into its related functions.

### TRQ affected the flagellar motility of *P. aeruginosa*

**Fig 3:**
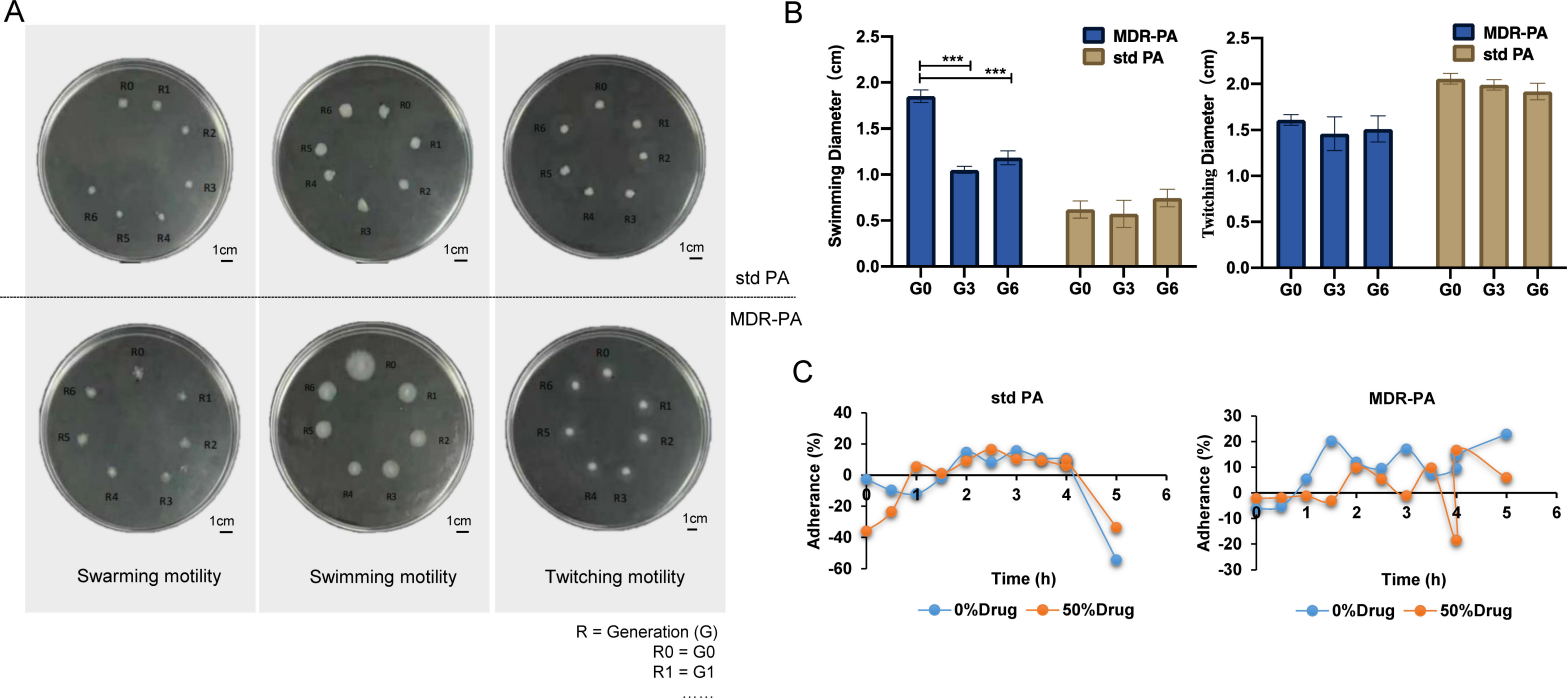
The motility and adhesion of MDR-PA after co-culture with TRQ. (**A and B**) The motility assay of *P. aeruginosa* after co-culture with TRQ. (**C**) The hydrophobicity assay of *P. aeruginosa* after co-culture with TRQ. The data of the bacterial circle diameter were expressed as means ± standard deviation and were analyzed by one-way analysis of variance (ANOVA) and Tukey’s multiple-comparison test; ***P＜0.001.

Flagellar motility is a crucial factor associated with the virulence of *P. aeruginosa*, as it plays a pivotal role in bacterial movement, colonization, biofilm formation, and adhesion to substrates. To assess the impact of TRQ on motility of *P. aeruginosa*, we conducted tests to evaluate swarming, swimming, and twitching motility (Fig. 3A and B). Following continuous co-culture of TRQ with bacteria, swimming motility exhibited a significant decrease in the diameter of the MDR-PA2 colony from 1.8 cm to 1.2 cm (*P* < 0.001). In swarming motility, the MDR-PA1 (G0 = R0) colony exhibited radial growth, which disappeared after continuous co-culture with TRQ. In twitching motility, for the MDR-PA2 colony, the diameter of the diffuse bacterial zone decreased from 1.6 cm to 1.5 cm from G0 to G6 (*P* > 0.05). The motility assay suggested that TRQ may impact the flagellar motility of *P. aeruginosa*.

### TRQ reduced the adhesion of MDR-PA

The adhesion rate of MDR-PA2 decreased significantly overall after TRQ treatment, especially after 4 h (Fig. 3C). This observation suggested that TRQ treatment could reduce the surface hydrophobicity of MDR-PA, resulting in weakened adhesion ability. This weakening of adhesion ability may not be conducive to its local colonization and could potentially reduce its pathogenicity.

### Co-immunoprecipitation and proteome analysis of *P. aeruginosa* after TRQ treatment

**Fig 4:**
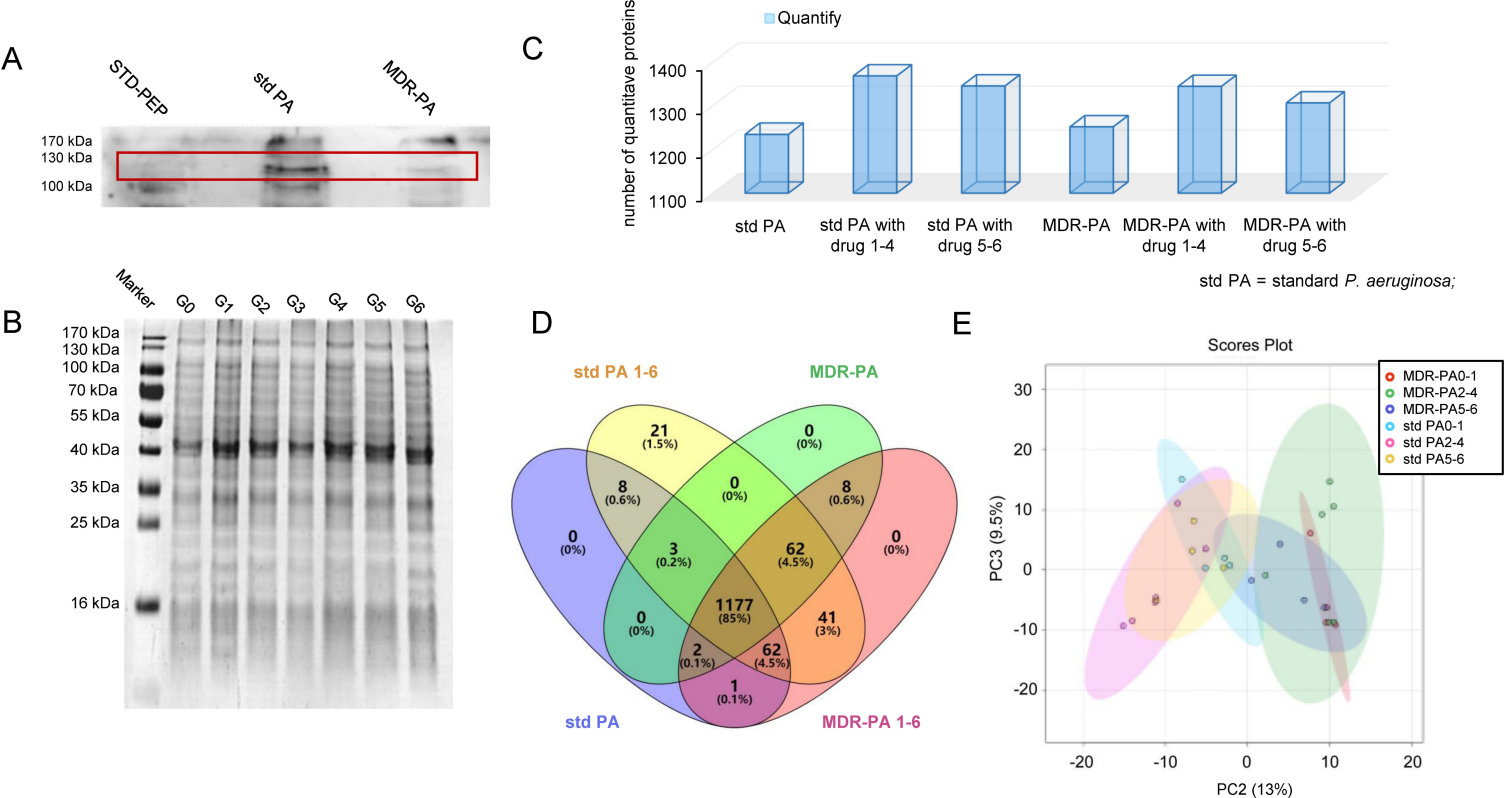
The identification and quantitative and bioinformatic analysis of the proteome. (**A**) Expression of immune antibody in standard *P. aeruginosa* (std PA) and MDR-PA bacterial proteins by Western blot. (**B**) The MDR-PA protein expression after co-culture with TRQ for six generations. (**C**) The number of label-free quantified proteins in std PA and MDR-PA group by Co-IP. (**D**) Overlap of highly confident proteins identified between the different *P. aeruginosa* groups (std PA, MDR-PA, std PA1-6, and MDR-PA1-6) for proteome. (**E**) The principal component analysis (PCA) between the different groups (std PA0-1, std PA2-4, std PA5-6, MDR-PA0-1, MDR-PA2-4, and MDR-PA5-6) after TRQ co-culture.

**Fig 5:**
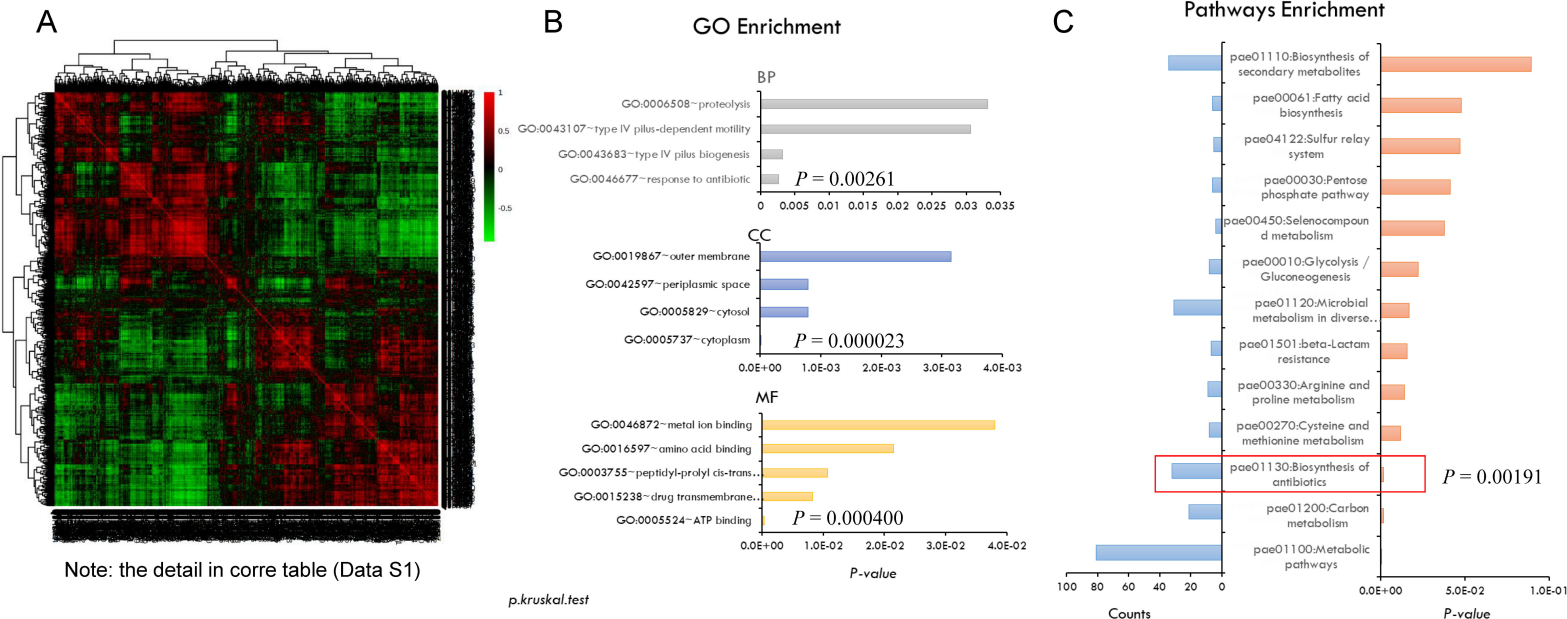
Bioinformatic analysis of the proteins with significantly altered expression in the proteome. (**A**) Clustering analysis of standard *P. aeruginosa* (std PA) and MDR-PA proteins using proteomic data (all the proteins in the x- and y-axis are detailed in Data S1). (**B**) The enrichment of biological processes for significantly altered proteins in the proteome of the std PA and MDR-PA by go annotation. (**C**) The enriched biological pathways for significantly altered proteins in the proteome of the std PA and MDR-PA by Kyoto Encyclopedia of Genes and Genomes (KEGG) pathway analysis. The *P* value was calculated using the hypergeometric distribution method.

We synthesized the Seq-PA No. 1 antigen and obtained antibody serum through mouse immunization. Antibody titer in the sera of immunized mice was 1:1,000, and the obtained antibody was subsequently purified. We detected the expression of the Seq-PA No. 1 peptide in standard *P. aeruginosa* and MDR-PA by Western blotting. The bands showed that the antibody was expressed in both bacterial proteins of standard *P. aeruginosa* and MDR-PA, with higher expression in the standard *P. aeruginosa* (Fig. 4A). Next, total protein lysates of standard *P. aeruginosa* and MDR-PA co-cultured with TRQ were separated by SDS-PAGE, but no obvious visual changes were observed in the electrophoresis bands (Fig. 4B). To identify differentially expressed proteins between standard *P. aeruginosa* and MDR-PA after TRQ treatment, Co-IP was performed using the Seq-PA No. 1 antibody, followed by in-depth proteome identification to analyze the protein interactions. Among these identified proteins, 1,177 (~85%) were common between standard *P. aeruginosa* and MDR-PA (Fig. 4C and D). Principal component analysis (PCA) was then performed, and the data of standard *P. aeruginosa* and MDR-PA after co-culture with TRQ for six generations were divided into three groups (0–1 generation, 2–4 generations, and 5–6 generations). The PCA results showed a certain degree of differentiation between the 0–1 and 2–4 generation groups, but the proteins of 5–6 generations showed similar expression between standard *P. aeruginosa* and MDR-PA (Fig. 4E). These findings indicated that the trend of drug resistance of MDR-PA was partially altered by successive generations of TRQ co-cultured. Through differential protein clustering analysis and correlation analysis, we discovered that the MDR-PA group and the standard *P. aeruginosa* group could be effectively distinguished from one another (Fig. 5A).

### KEGG pathway enrichment and Gene Ontology (GO) enrichment of the significantly altered proteins

The function of differentially expressed proteins was analyzed using GO enrichment analysis. The most significant *P* value among these proteins associated with the biological process was the antibiotic response (GO: 0046677 ~ response to antibiotic, *P* = 0.00261), while the cellular component found significant was the cytoplasm (GO: 005737 ~ cytoplasm, *P* = 0.000023), and in terms of molecular function, the primary association was with ATP binding (GO: 005524 ~ ATP binding, *P* = 0.000400) (Fig. 5B). Our study concentrated on exploring the “antibiotic response.” The KEGG pathway enrichment analysis revealed a significantly enriched “biosynthesis of antibiotic” pathway (*P* = 0.00191) (Fig. 5C). The functional proteins were obtained (Table S2), which were closely related to antibiotic resistance, cell division, biosynthetic pathways, and other relevant processes, and further verification needs to be performed.

### qPCR validation for these candidates

**Fig 6:**
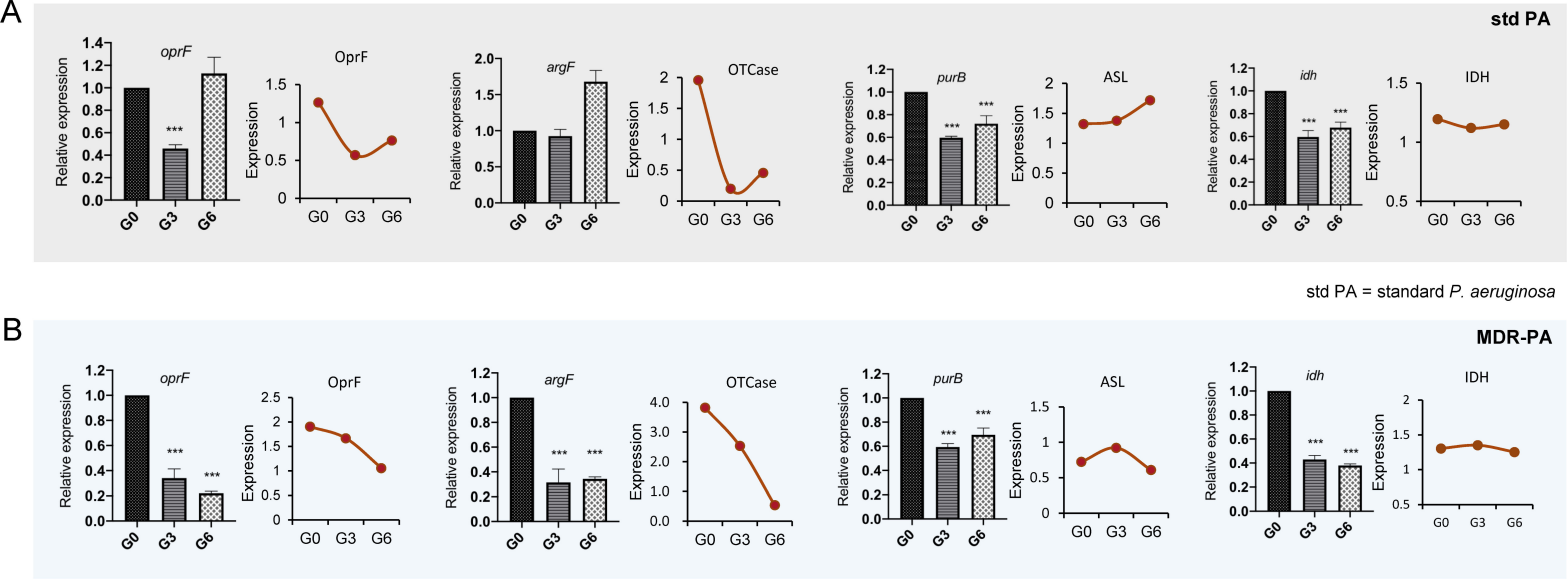
Selection and validation of the core candidates from STRING by qPCR. (**A**) The protein and gene expression of the core candidates for the standard *P. aeruginosa* (std PA). (**B**) The protein and gene expression of the core candidates for the MDR-PA. The data of the gene expression were expressed as means ± standard deviation and were analyzed by one-way ANOVA and Tukey’s multiple-comparison test; ***P＜0.001.

**Fig 7:**
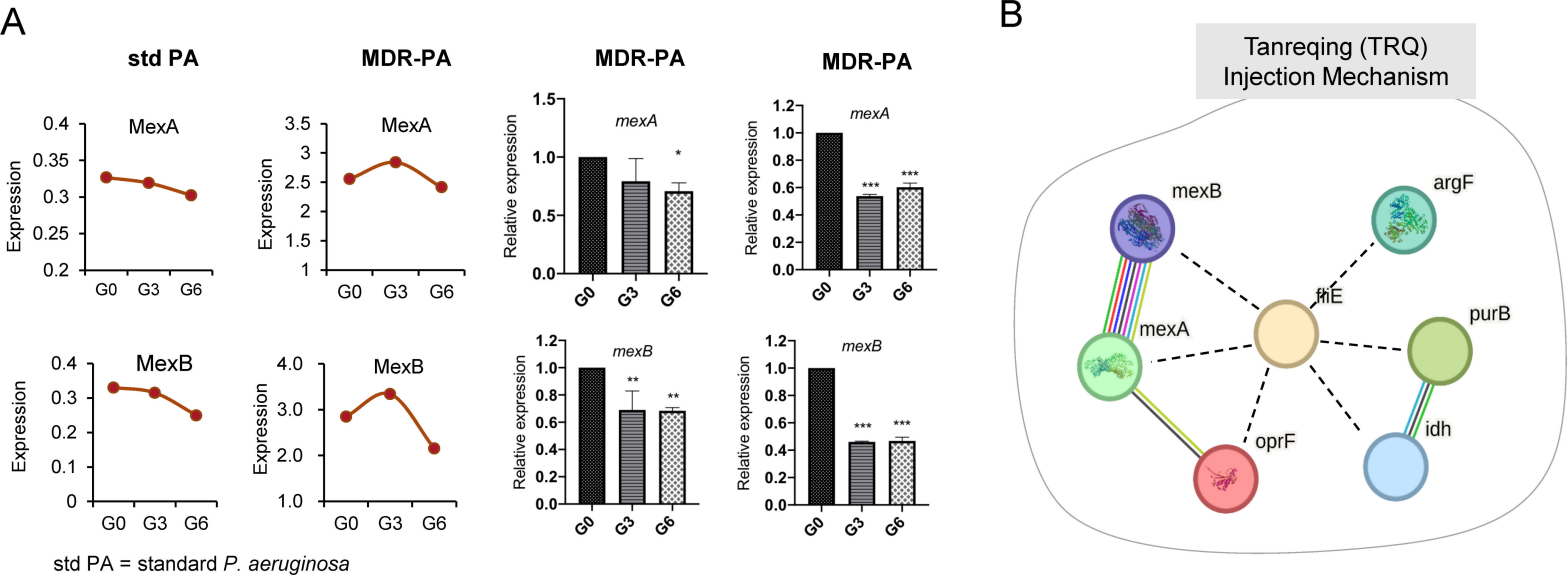
Another selection and validation of two candidates by qPCR. (**A**) The protein and gene expression of MexA and MexB associated with efflux pump for the standard *P. aeruginosa* (std PA) and MDR-PA. (**B**) Hierarchical cluster result of the network based on STRING clustering analysis. The data of the gene expression were expressed as means ± standard deviation and were analyzed by one-way ANOVA and Tukey’s multiple-comparison test; ***P＜0.001; **P＜0.01; *P＜0.05.

To further validate the sensitizing effects of TRQ, we performed qPCR verification on different generations (G0, G3, and G6) of standard *P. aeruginosa* and MDR-PA strains after co-culture with TRQ. Results confirmed that genes related to antibiotic resistance, cell division, biosynthetic pathways, and other processes were significantly downregulated in MDR-PA5 and MDR-PA7 strains, which is consistent with proteomic analysis. The genes *mexA* (*P* < 0.05), *mexB* (*P* < 0.01), *oprF* (*P* < 0.001), and *idH* (*P* < 0.01) in MDR-PA5 strain and *mexA* (*P* < 0.001), *mexB* (*P* < 0.001), *oprF* (*P* < 0.001), *idH* (*P* < 0.001), *oprM* (*P* < 0.001), *purB* (*P* < 0.001), and *argF* (*P* < 0.001) in MDR-PA7 strain were significantly downregulated. The genes (*oprF*, *purB*, *argF*, and *idH*) and corresponding proteins (OprF, ASL, OTCase, and IDH) in standard *P. aeruginosa* and MDR-PA were compared (Fig. 6A and B). Notably, both genes (*mexA* and *mexB*) and proteins (MexA and MexB) were downregulated in standard *P. aeruginosa* and MDR-PA. This suggested an association with the efflux pump MexAB-OprM in *P. aeruginosa* (Fig. 7A and B). Additionally, the down-regulation of outer membrane porin F (OprF), which plays roles in cell shape, ion transport, and transport, revealed that TRQ may sensitize antibiotic resistance through these mechanisms.

## DISCUSSION

In this study, we aimed to investigate the antibacterial effect of TRQ on clinical MDR-PA and elucidate the associated pharmacological mechanisms. We employed multiple methods to explore the main targets of TRQ in overcoming resistance of *P. aeruginosa*. For instance, MALDI-TOF-MS detection and Biotyper evaluation scores in Fig. 2B revealed an increasing confidence level of MDR-PA after co-culture with TRQ for six generations. Furthermore, the results of resistance trend of MDR-PA with cefoperazone and ceftazidime were decreased by traditional MIC measurement in Fig. 2A. These findings indicated that TRQ may affect the resistance of MDR-PA. We utilized protein fingerprints to identify a differential sequence, Seq-PA No. 1, which was associated with the motility and attachment of flagella. We then conducted motility assays and the bacterial cell surface hydrophobicity of *P. aeruginosa*, as shown in Fig. 3. The results revealed that TRQ could impact flagellar motility and reduce the surface hydrophobicity of MDR-PA. These observations align with previous reports (33). Additionally, we employed the Co-IP method to identify proteins that interacted with Seq-PA No. 1 in standard *P. aeruginosa* and MDR-PA before and after TRQ co-culture. Furthermore, we analyzed the core proteins through proteome analysis, as depicted in Fig. 5, which aided in further elucidating the mechanism through which TRQ can affect drug resistance in *P. aeruginosa*.

Using MALDI-TOF-MS, we obtained the confidence scores and MALDI fingerprints (as shown in Fig. 2B through D). We compared MALDI fingerprint with Seq-PA No. 1 sequence using the Mascot search engine. If the MALDI fingerprint does not match Seq-PA No. 1, the *P. aeruginosa* to be tested is a candidate for drug resistance strain. Conversely, if the MALDI fingerprint matches Seq-PA No. 1, the *P. aeruginosa* to be tested is not a candidate for drug resistance strain. The method is easy to operate and can quickly and accurately identify drug-resistant or drug-sensitive bacteria. The entire process takes less than 10 minutes, offering the advantages of being less time consuming, highly sensitive, and specific. This efficiency can save valuable time for the treatment of severe infections caused by this type of bacteria in clinical settings.

β-Lactams, aminoglycosides, and fluoroquinolones are commonly used antibiotics in the clinical management of *P. aeruginosa* infections. However, resistance to these antibiotics can lead to treatment failures in infections caused by MDR-PA (34). Efflux pumps, such as the RND family efflux pumps, can lead to antibiotic resistance in *P. aeruginosa*. RND efflux pumps, such as MexAB-OprM, MexCD-OprJ, MexEF-OprN, and MexXY-OprM, are commonly associated with antibiotic resistance in *P. aeruginosa* (13). Among these, the MexAB-OprM efflux system is a prominent resistance mechanism in *P. aeruginosa*, and hyper-expression of this system has been reported (35). The MexAB-OprM efflux system is composed of a periplasmic membrane fusion protein (MexA), an inner membrane drug-proton antiporter (MexB), and an outer membrane channel-forming component (OprM) (36). The tripartite multidrug efflux system MexAB-OprM utilizes proton gradient as a driving force to pump out multiple antibiotics from the bacterial cell. The outer membrane barrier interacts with the MexAB-OprM efflux system to facilitate the extrusion of substrates. The substrates either from the lipid bilayer or from cytoplasmic space are recognized and captured by MexB; then, a channel in the outer membrane is formed, and the substrates are transported to the extracellular medium through OprM. MexA plays a coordinating role in the cooperation between MexB and OprM. Efflux pump inhibitors have been found to restore bacterial susceptibility to multiple drugs (37, 38). In our study, as illustrated in Fig. 4 and 5, we conducted Co-IP and proteome analysis, which revealed a decrease in the expression of MexA and MexB at the protein level. This finding was further confirmed by a consistent downward trend at the gene level, as demonstrated in Fig. 7. These results suggested that TRQ might regulate the expression of the MexAB-OprM efflux pump in MDR-PA. MexAB-OprM is primarily responsible for extruding β-lactams and quinolones antibiotics. As shown in Fig. 2A; Table S1, the MICs of cefoperazone and ceftazidime, both of which are β-lactam antibiotics, tended to decrease after TRQ treatment. This phenomenon may explain why TRQ can reduce the resistance of MDR-PA to ceftazidime and cefoperazone.

*P. aeruginosa* resistance is also linked to various porins that can affect the penetration of antibiotics. OprF, the predominant porin in *P. aeruginosa*, exhibits low efficiency in allowing antibiotics to penetrate the bacterial cell. OprF exists in two conformers; the dominant structure of OprF channels is a closed conformer, and less than 5% is an open conformer (39). Higher levels of OprF in the outer membrane of *P. aeruginosa* are accompanied by thick biofilms, which are closely related to drug resistance (40). In this study, co-culturing clinical MDR-PA with TRQ for six generations led to downregulation of OprF protein expression, as shown in Fig. 6. In Fig. 3C, we also observed a significant reduction in the adhesion rate after TRQ treatment. This suggested that TRQ might be effective in reducing the resistance of MDR-PA. Researchers have found that the adhesion of *P. aeruginosa* to human lung alveolar epithelial cells was at least partially mediated by OprF due to a significant reduction in the binding capacity of *oprF*-negative mutants (40).

*P. aeruginosa* has EC 2.1.3.3 and ornithine carbamoyl transferases (OTCases). The anabolic OTCase catalyzes the sixth reaction in arginine biosynthesis, which is the product of the *argF* gene. The catabolic OTCase is the product of the *arcB* gene and is related to anaerobic arginine catabolism. Both enzymes function unidirectionally in *P. aeruginosa*; no matter *arcB* or *argF* is mutated, the anabolic or catabolic metabolism will be affected (41). IDH (*idh*) is relevant to the tricarboxylic acid (TCA) cycle–glyoxylate shunt branch point in *P. aeruginosa. P. aeruginosa* mutually regulates ICL and IDH through oxaloacetate and pyruvate, thereby coordinating the allocation of metabolic flux (42). The ASL (*purB*) plays a crucial role in catalyzing two reactions within the *de novo* purine nucleotide biosynthesis process. IDH, ASL, and OTCase (*argF*) mainly affect bacterial biosynthesis pathway and bacterial growth, and TRQ can also downregulate the expression of three proteins above as shown in Fig. 6. In addition, it is the first time for our study to report that these proteins (IDH, OTCase, and ASL) were associated with the resistance synthesis of MDR-PA. It was hypothesized that TRQ changed drug resistance by affecting the biosynthesis of *P. aeruginosa*.

### Conclusion

In this study, a simple and reliable phenotypic identification method was established to reflect the clinical drug resistance and sensitivity of *P. aeruginosa* by comparing the fingerprint and the characteristic amino acid sequence of the strains. We applied this method to real clinical samples, showcasing its rapidity, sensitivity, and reliability. Simultaneously, we integrated Co-IP and proteomics and performed PCR validation to investigate the mechanism of clinical MDR-PA resistance and the pharmacological effects of TRQ on core proteins (MexA, MexB, OprM, OprF, OTCase, IDH, and ASL) associated with *P. aeruginosa* resistance. This is the first time that we report the mechanism by which TRQ re-sensitizes drug-resistant *P. aeruginosa* and the specific targets of TRQ. The integration of multiple methods offers a new direction for screening and developing new drugs and mining drug resistance mechanisms and action targets. This approach holds great promise for applications in the detection of drug-resistant bacteria and beyond.

## MATERIALS AND METHODS

### Bacterial strains and culture conditions

*P. aeruginosa* (ATCC 27853) was purchased from the American Type Culture Collection (ATCC, USA). Multidrug-resistant *Pseudomonas aeruginosa* isolates were obtained from the clinical laboratory of Wangjing Hospital, Beijing, China. All maintained in cryogenic storage at −80°C. Strains were grown in lysogeny broth (LB) or Mueller-Hinton broth (MHB) at 37°C under standard storage conditions. The LB and agar were used for reversal of drug resistance and the MHB and agar were used for AST. Azlocillin, cefoperazone, and ceftazidime antibiotics were selected for AST assay.

### Animal preparation

Eight-week-old C57BL/6 male mice weighing 19–21 g were obtained from Beijing Weitong Lihua Laboratory Animal Technology Co., Ltd., China. Every five mice were kept in a cage with free access to food and water; all animals were housed in an environment with a relative humidity of 50% ± 10% and a temperature of 22°C ± 2°C, along with a 12-h light/12-h dark cycle.

### MIC assays

The broth dilution assays were used to determine the MICs as described previously (43, 44). Briefly, 5 × 10^5^ CFU/mL bacterial suspension was incubated in 96-well plates with varying concentrations of antibiotics. After incubation at 37°C for 18–24 h, the MICs of *P. aeruginosa* were determined before and after TRQ co-cultivation.

### Co-culture of TRQ and MDR-PA

*P. aeruginosa* were incubated at 37°C in a 1:1 ratio containing TRQ and LB medium and shaken at 280 rpm for 18–20 h. Overnight-grown bacterial suspensions were diluted with MHB to 10^5^ CFU/mL for MIC assays. After incubation, the bacteria suspension was streaked over the surface LB agar at 37°C for 16–20 h under aerobic condition and recorded as the first generation. The operation was repeated in the sixth generation, and 14 strains were obtained and stored at 4°C.

### Identification of bacterial successive passaged by MALDI-TOF-MS

*P. aeruginosa* colonies were extracted using formic acid and then subjected to identification using MALDI-TOF-MS (Bruker Daltonics, Bremen, Germany) (45). A certain amount (5–10 mg) of purified colony was taken from the plate and suspended in 300-µL purified water by vortexing and was mixed carefully by adding 900-µL absolute ethanol (after this treatment, the sample can be stored at −20°C for several weeks). Then, it was centrifuged at 13,000 rpm for 2 min, and the supernatant was discarded (if necessary, it was centrifuged again to remove ethanol as much as possible); 70% formic acid (50 µL) was added and the precipitate was suspended and then mixed by adding acetonitrile (50 µL) and centrifuged for 2 min at a high speed. Then, the supernatant (1 µL) was placed on a MALDI-TOF MS steel anchor plate (BigAnchor 384-well plate; Bruker Daltonics) and dried. The dried mixture was covered with the saturated α-cyano-4-hydroxycinnamic acid matrix solution (HCCA, 1 µL, Bruker Daltonics) and dried prior to analysis using MALDI Biotyper. To avoid protein oxidation, the operation should be completed as soon as possible, especially when adding the matrix solution. Acquisition parameters were as follows: the shots were 500, the scan range was 2,000–20,000 m/z, N2 laser wavelength was 337 nm, and laser energy was 85%. Bruker Biotyper 3.0 software was used to analyze the spectra. Identification was carried out using the manufacturer’s recommended and internally determined cutoff scores; the protein identification and analysis were carried out by mass spectrometry of peptides using MALDI-TOF-MS (46).

### Swimming motility assay

LB agar (0.3%) plates were used to assess the motility of *P. aeruginosa* mediated by flagellum. The overnight LB cultures (aliquots, 1 µL) were inoculated in agar, and the diameter of the swimming zone was measured after 16-h incubation at 30°C. All assays were performed in triplicate (47, 48).

### Twitching motility assay

*P. aeruginosa* isolates were inoculated using a needle placed at the bottom of the plates containing 3 mm deep 1% LB agar, followed by incubation at 37°C for 24 hours. Subsequently, the twitch zone between the agar and the plate bottom was measured. All assays were performed in triplicate (49, 50).

### Swarming motility assay

The swarming motility of the isolates was tested in plates (0.5% agar, 0.05% monosodium glutamate, and 0.2% glucose). The overnight LB cultures (aliquots, 1 µL) were inoculated on the agar surface; after incubating 24 h at 30°C, the radial growth of swarming was observed. All assays were performed in triplicate (51, 52).

### Hydrophobicity assay

The adhesion to n-hexadecane was used to determine the relative surface hydrophobicity of *P. aeruginosa* by TRQ treatment. Briefly, the bacteria were cultured overnight in the logarithmic phase and diluted with a 1:1 ratio containing TRQ and LB medium or only in LB medium at 37°C and shaken at 100 rpm for 5 h. Then, cultures (4 mL) were taken every 30 min and centrifuged at 4,700 rpm for 5 min. The precipitate was washed twice with PBS and suspended in 3-mL PBS, and 0.8 mL of n-hexadecane was added. Then, the mixture was vortexed for 30 s. Finally, 1-mL n-hexadecane phase was taken, and the absorbance was measured at 540 nm. The results were determined by the following equation: % adherence = [(1 − A/A0)] × 100, where A0 was the initial optical densities of the aqueous phase and A was the final optical densities of the aqueous phase (53, 54). .

### Preparation of mouse polyclonal antibody by immunized mice

Before immunization, antigenic polypeptide (Seq-PA No. 1, 1 mL) was emulsified with Freund’s adjuvant (1 mL). Then, Seq-PA No. 1 (100 μg) was intraperitoneally injected into C57BL/6 male mice every 2 weeks for a total of eight to nine times. Before and after immunization, blood samples (1.5–2.0 mL) were collected from the tail of the mice to obtain polyclonal antibody serum. The antibody titers in the immunized mice were determined by enzyme linked immunosorbent assay (ELISA).

### Purification of mouse polyclonal antibody

The antibody was purified using a protein A column. After rinsing with PBS, the antibody was permeabilized with 0.1 M citric acid for 5 min, followed by thorough washing, repeated three times. Neutralization was conducted with 1 M Tris-HCl (pH 8.0) and PBS equilibrated, followed by extensive washes with a 20% ethanol solution. The upper and lower ends were sealed and stored at 4°C. The specificity of the antibody was determined by SDS-PAGE and Western blot (55).

### Extraction and quantification of *Pseudomonas aeruginosa* protein

After centrifugation at 5,000 × *g* for 10 min at 4°C, the bacterial culture precipitate was resuspended in 1-mL PBS, then centrifuged again at 15,000 rpm for 3 min, and washed three times at 4°C. The proteins were extracted in a mixture containing sample, urea, and protease inhibitor (5 mg:15 µL:1 µL ratio). The total bacterial protein was extracted using ultrasonic crushing for 20 s on ice. Total protein content was determined using a BCA protein assay kit (Thermo Scientific, Inc.).

### Western blotting

As previously described, we conducted Western blot analysis. Standard *P. aeruginosa* and MDR-PA proteins were separated on a SDS-PAGE. After electrophoresis, we performed wet transfer, incubating the membrane at 4°C for 75 min at 100 V, followed by blocking with skimmed milk powder for 1 h. The membranes were then incubated with primary antibodies (the purified antibody was used as the primary antibody at a 1:1,000 dilution, and β-actin served as the internal reference antibody at a 1:2,500 dilution) at 4°C for 3 h. Subsequently, the membranes were washed in TBST for 10 min, four times in total, and the secondary antibody (1:5,000) was added and incubated at 4°C for 1 h. The membranes were washed four times in TBST. The quantification of blot was conducted using ImageJ software (V1.8.0) for grayscale analysis (55).

### Co-IP assay

After ultrasonic extraction as described previously, 200 µL of protein was taken from each sample. To one tube, 10 µL of antibody-containing serum was added, and to the other tube, 10 µL of antibody-free serum was added. After sealing, the samples were incubated at 4°C for 24 h. Then, 100 µL of protein A/G beads was added, and the samples were incubated for 2 h with shaking. The precipitate was collected by centrifugation at 1,000 × *g* for 1 min and washed five times with PBS (56).

### Trypsin digestion

In-solution tryptic digestion was conducted following the previously described method (57). An 8 M urea was used to denature the protein obtained by immunoprecipitation, and 10 mM dithiothreitol and 55 mM iodoacetamide were used to reduce and alkylate the proteins. The sample solution was then diluted with 50 mM NH_4_HCO_3_ to reduce the urea concentration to 1 M. The proteins were digested overnight at 37°C in a trypsin-to-protein ratio of 1:100, and the reaction was stopped by adding a final concentration of 0.1% formic acid (FA). The supernatant was collected.

### Desalination by C18 column

The C18 column was washed twice with acetonitrile (ACN) and 0.1% formic acid (FA), respectively. The supernatant obtained earlier was slowly added to the column, and the effluent was collected, repeating this step three times. Afterward, the column was washed twice with 0.1% FA. The target components were eluted using a mixed solution of 50% ACN and 0.1% FA, and the eluent was collected for mass spectrometry detection.

### Proteomic identification and quantification of Co-IP proteins

For the LC-MS/MS analysis of peptides, a QExactive-HFX mass spectrometer (Thermo Fisher, USA) equipped with an EASY-nano-1000 LC system (Thermo Fisher, USA) and a nanoelectrospray ion source (Thermo Fisher, USA) was employed (58). A 5-µL sample was injected into a self-packed precolumn (20 mm, 5 µm, 100 A) at a flow rate of 300 nL/min. Separation was achieved using a self-packed reversed-phase C18 nano-column, with mobile phase A consisting of 0.1% formic acid in water and mobile phase B containing 0.1% formic acid in 80% acetonitrile. The mass scan ranged from 300 to 1,400 m/z, with a resolution of 60,000, and the 20 most intense ions were sequentially separated. The electrospray voltage was 1.9 kV, and the capillary temperature was maintained at 320°C.

The fragmentation spectra were searched using the Mascot search engine (v 2.2.06), with the Uniport *P. aeruginosa* database (20190320). For the QE data, the precursor and fragment mass tolerances were set to 10 ppm and 20 mmu, respectively. There were two missed cleavage sites, and the minimum peptide length was set to seven amino acids. The fixed and variable modifications included carbamidomethyl (C), acetylation (protein N-terminal), and oxidation (M). Peptide ions were filtered using cutoff scores from percolators (*P* < 0.01), ensuring a false discovery rate of 1% for peptide identifications. The Proteome Discoverer 2.3 proteomic platform was used to process the raw data, and label-free quantitative analysis of proteins was performed using an in-house software (FileMaker).

### RNA isolation and RT-qPCR

Total bacterial RNA was extracted following the manufacturer’s protocol (Tiangen Biotec, China), and the concentrations of RNA were measured using NanoDrop (Thermo Fisher, USA). ReverTra Ace qPCR RT Master Mix (Toyobo, Japan) was used to synthesize complementary DNA, and mRNA quantification was performed using SYBR Green Real-time PCR Master Mix (20-µL reaction volume, Toyobo, Japan). The *rpoD* was used as a reference gene. Data analysis was performed using the comparative threshold cycle (2^−∆∆^Cq) method, and qPCR primers were obtained from Sangon Biotech (Shanghai) Co., Ltd.

### Bioinformatic analysis

Gene Ontology annotation (https://geneontology.org) (59), DAVID Bioinformatics Resources (https://david.ncifcrf.gov/) (60), and STRING database (https://cn.stringdb.org/) (61) were employed to explore the subcellular localizations, biological functions, related networks, and pathways of the identified significant GPs. Statistical Product and Service Solutions (SPSS 19.0) software (IBM Corporation, Armonk, New York, USA) was used to analyze the remarkably regulated proteins and gene symbols. The *P* value was calculated using the hypergeometric distribution method.

## Data Availability

The mass spectrometry proteomic data have been deposited to the ProteomeXchange Consortium (http://proteomecentral.proteomexchange.org) via the iProX partner repository (62, 63) with the data set identifier PXD039911.

## SUPPLEMENTAL MATERIAL

Data S1 spectrum.02726-23-s0001.xlsx Data for Fig. 5A.

Tables S1 and S2 spectrum.02726-23-s0002.pdf Supplemental tables.

ASM does not own the copyrights to Supplemental Material that may be linked to, or accessed through, an article. The authors have granted ASM a non-exclusive, world-wide license to publish the Supplemental Material files. Please contact the corresponding author directly for reuse.
