# Supplementary material for: In-depth analysis of the treatment effect and synergistic mechanism of TanReQing injection on clinical multi-drug resistant Pseudomonas aeruginosa
Source: Microbiol Spectr. 2024 Feb 28;12(4):e02726-23. doi: 10.1128/spectrum.02726-23 (PMC10986576; doi:10.1128/spectrum.02726-23)
Supplement: Tables S1 and S2 — Supplemental tables. [file spectrum.02726-23-s0002.pdf]

Supplementary Material

In-depth analysis of the treatment effect and synergistic mechanism of TanReQing injection on clinical multi-drug resistant *Pseudomonas aeruginosa*

Dongying Li, Yueyi Li, Jingyi Wang, Weifeng Yang, Kaiyu Cui, Renjing Su, Lu Li, Xing Ren, Xianyu Li*, Yi Wang*

1. Supplementary Tables

Table S1 Antibiotic MIC values after TRQ and MDR-PA co-culture for six generations

Antibiotics	strains	antibiotic MICs (μg/ml)						
Antibiotics	strains	G0	G1	G2	G3	G4	G5	G6
Azlocillin	PA27853	8192	256	128	512	256	256	256
	MDR-PA7	8192	128	128	64	64	128	128
Cefoperazone	PA27853	16	16	16	16	16	16	16
	MDR-PA1	8192	8192	8192	8192	8192	8192	1024
	MDR-PA9	8192	8192	8192	8192	8192	2048	8192
	MDR-PA7	8192	8192	16	32	32	16	32
	MDR-PA8	8192	8192	4096	4096	2048	4096	1024
Ceftazidime	PA27853	4	4	4	4	4	4	4
	MDR-PA2	4096	1024	2048	2048	2048	2048	1024
	MDR-PA5	8192	8192	8192	8192	4096	2048	2048

Table S2 Obtained functional protein by Co-immunoprecipitation and GO analysis

Name	Protein	gene	Biological process
Q51487	Outer membrane protein OprM	*oprM*	Antibiotic resistance, Transport
Q9HWR2	Aminoglycoside 3'-phosphotransferase	*aph*	Antibiotic resistance
G3XCW2	Efflux pump membrane transporter	*PA2018*	Cell cycle, Cell division
P52477	Multidrug resistance protein MexA	*mexA*	Antibiotic resistance, Transport
P52002	Multidrug resistance protein MexB	*mexB*	Antibiotic resistance, Transport
G3XD21	Resistance-Nodulation-Cell Division (RND) multidrug efflux membrane fusion protein	*PA2019*	Cell cycle, Cell division
Q9HW02	UDP-N-acetylmuramate--L-alanine ligase	*murC*	Cell cycle, Cell division, Cell shape, Cell wall biogenesis/degradation, Peptidoglycan synthesis
P13794	Outer membrane porin F	*oprF*	Cell shape, Ion transport, Transport
P43336	Aromatic-amino-acid aminotransferase	*phhC*	Amino-acid biosynthesis, Aromatic amino acid biosynthesis
P11724	Ornithine carbamoyltransferase, anabolic	*argF*	Amino-acid biosynthesis, Arginine biosynthesis
Q9HVA0	Acetolactate synthase	*ilvI*	mino-acid biosynthesis, Branched-chain

			amino acid biosynthesis
Q9I0K9	Adenylosuccinate lyase	*purB*	Purine biosynthesis
P42805	4-diphosphocytidyl-2-C-methyl-D-erythritol kinase	*ispE*	Isoprene biosynthesis
Q9I6E0	Dihydroxy-acid dehydratase	*ilvD*	Amino-acid biosynthesis, branched-chain amino acid biosynthesis
O30508	Succinylornithine transaminase/acetylornithine aminotransferase	*aruC*	Amino-acid biosynthesis, Arginine biosynthesis, Arginine metabolism
Q9I0L4	Isocitrate dehydrogenase [NADP]	*idh*	Glyoxylate bypass；Tricarboxylic acid cycle
